# Atypical Appendicitis Presenting as Left Upper Quadrant Pain: A Case Report

**DOI:** 10.7759/cureus.100026

**Published:** 2025-12-24

**Authors:** Elisabeth Hildenbrandt, Mark E Hoffman, Mark Salib, John Salib, Frederick Tiesenga

**Affiliations:** 1 School of Medicine, St. George’s University School of Medicine, True Blue, GRD; 2 General Surgery, West Suburban Medical Center, Chicago, USA

**Keywords:** acute appendicitis, appendectomy variants, atypical appendicitis, ct imaging, diagnosing appendicitis, diagnosis of acute appendicitis, general surgery, laparascopic surgery, laparoscopic appendectomy, retrocecal appendicitis

## Abstract

Acute appendicitis persists as one of the leading indications for emergency abdominal surgical intervention globally. Although its classic presentation is well described, atypical manifestations continue to present diagnostic difficulties and may contribute to delays in treatment and higher rates of complications. We describe a case involving a 21-year-old woman with no notable past medical history who presented with a six-hour history of left upper quadrant abdominal pain accompanied by nausea and anorexia. On examination, she exhibited localized abdominal tenderness without evidence of guarding or rebound. CT imaging of the abdomen and pelvis demonstrated an unusually elongated, retrocecal appendix extending superiorly, containing an obstructing appendicolith with surrounding inflammatory changes, findings consistent with acute appendicitis. The patient subsequently underwent an emergent laparoscopic appendectomy without complication. This case highlights the need for continued clinical vigilance when evaluating abdominal pain with nontraditional localization. In the presence of atypical symptoms, early cross-sectional imaging plays a crucial role in establishing a diagnosis, particularly in patients with anatomical variants, and may help reduce delays in definitive surgical intervention.

## Introduction

Acute appendicitis is among the most frequent causes of abdominal surgical emergencies worldwide, with an estimated incidence of approximately 100 cases per 100,000 individuals annually [1]. It is often introduced as a clinically straightforward diagnosis, classically described as periumbilical pain that migrates to the right lower quadrant and is accompanied by nausea, vomiting, anorexia, and focal tenderness at McBurney’s point [1,2]. In practice, however, this pattern is far from universal. Variations in the position and length of the appendix can substantially alter symptom localization and undermine reliance on traditional teaching alone [1,3].

When the appendix is retrocecal and elongated, inflammatory pain may be displaced away from the right lower quadrant and manifest in unexpected regions of the abdomen [2,4]. This anatomic configuration is particularly misleading in young patients who otherwise appear well and lack peritoneal signs on examination [1,3,5]. In such settings, the diagnosis may be overlooked or delayed, thereby increasing the likelihood of complications that could be avoided with timely intervention [4-6].

Cross-sectional imaging, therefore, assumes a central role in the evaluation of patients with atypical presentations. CT is especially valuable, not only because of its high diagnostic accuracy but also because it allows precise characterization of appendiceal anatomy and associated pathology [4,5]. Appendicitis most commonly occurs in individuals between the ages of 10 and 30 years [1-3], and laparoscopic appendectomy remains the definitive treatment in the majority of cases [7]. Although antibiotic therapy may be considered in selected patients with uncomplicated disease [5,7], early operative management is often favored when anatomic variation, luminal obstruction, or diagnostic uncertainty is present, as these factors may increase the risk of treatment failure or recurrence with conservative approaches [5-7].

Although right lower quadrant pain remains the most common presentation, atypical features are reported in a substantial proportion of patients [1,4,5]. We describe a case of acute appendicitis in a previously healthy young woman who presented with isolated left upper quadrant abdominal pain due to a retrocecal, elongated appendix containing an obstructing appendicolith [6,7]. This case illustrates the limitations of symptom-based diagnosis alone. It reinforces the importance of early imaging and sustained diagnostic vigilance when evaluating abdominal pain that localizes outside its expected distribution [6,8].

## Case presentation

A 21-year-old woman presented to the emergency department with a six-hour history of sharp, non-radiating pain localized to the left upper quadrant of the abdomen, which she rated as 7 out of 10 in severity. The pain was associated with anorexia and nausea without vomiting. She denied any history of trauma, right lower quadrant pain, diarrhea, constipation, dysuria, hematuria, chest pain, shortness of breath, cough, palpitations, fever, or chills. She reported active menstruation at the time of presentation. Her past medical and surgical histories were unremarkable. She reported no known drug allergies, and her social history was noncontributory.

On examination, the patient appeared mildly fatigued and uncomfortable due to abdominal pain but remained alert, oriented, and cooperative, responding appropriately to questions. She was afebrile and hemodynamically stable at the time of evaluation. The abdominal examination revealed a soft, nondistended abdomen with normal bowel sounds. Focal tenderness was present in the left upper quadrant and was reproduced with both superficial and deep palpation, without associated guarding or rebound tenderness. McBurney’s point was nontender, and Rovsing, obturator, and psoas signs were not elicited. No costovertebral angle tenderness, palpable masses, or hepatosplenomegaly were identified. Cardiopulmonary examination revealed a regular heart rate and rhythm, with normal heart sounds, and clear breath sounds bilaterally, without any adventitious findings. Considering the patient’s age, absence of chest pain, unremarkable cardiopulmonary examination, and lack of cardiovascular risk factors, further cardiac evaluation with ECG or serum troponin was deemed unnecessary.

Initial laboratory evaluation (as depicted in Table 1) revealed leukocytosis with a white blood cell count of 14.9 × 10^9^/L, driven by an absolute neutrophilia of 10.2 × 10^9^/L, consistent with an acute inflammatory process. Hemoglobin, hematocrit, platelet count, and red blood cell indices were within normal limits. The comprehensive metabolic panel was unremarkable, with preserved renal function, normal electrolytes, and no evidence of hepatic dysfunction. Serum lipase was within the normal range. A urine pregnancy test was negative.

**Table 1:** Laboratory results on admission. Initial laboratory evaluation on admission, including complete blood count with differential and comprehensive metabolic panel. Values are shown with corresponding reference ranges.

Section	Test name	Results	Reference range
Complete blood count (CBC)	White blood cells	14.9	4.0–11.0 k/mm³
	Red blood cells	4.04	3.63–5.04 m/mm³
	Hemoglobin	13	12–15.3 g/dL
	Hematocrit	38.6	34.7–45.1%
	Mean corpuscular hemoglobin	32.2	26–34 pg
	Mean corpuscular volume	95.6	80.0–100.0 fL
	Mean corpuscular hemoglobin concentration	33.7	32.5–35.8%
	Red cell distribution width	13.6	11.9–15.9%
	Platelets	379	150–450 k/mm³
	Mean platelet volume	7.7	6.8–10.2 fL
Complete blood count differential	Lymphocytes %	24.5	20–40%
	Monocytes %	6	2–8%
	Eosinophils %	1.2	1–4%
	Basophils %	0.1	0–1%
	Segmented neutrophils absolute	10.2	1.7–7.7 k/mm³
	Lymphocytes absolute	3.7	0.6–3.4 k/mm³
	Monocytes absolute	0.9	0.3–1 k/mm³
	Eosinophils absolute	0.2	0–0.5 k/mm³
	Basophils absolute	0	0–0.2 k/mm³
Complete metabolic panel	Total protein	7.4	6.0–8.3 g/dL
	Glucose	95	70–99 mg/dL
	Blood urea nitrogen	10	7–20 mg/dL
	Creatinine	0.71	0.6–1.3 mg/dL
	Sodium	139	135–145 mmol/L
	Potassium	3.5	3.5–5.1 mmol/L
	Chloride	105	98–107 mmol/L
	Carbon dioxide	24	22–29 mmol/L
	Anion gap	10	8–16 mmol/L
	Blood urea nitrogen/Creatinine ratio	14	10–20
	Calcium	9.6	8.5–10.5 mg/dL
	Albumin	4.8	3.5–5.5 g/dL
	Aspartate aminotransferase	14	10–40 U/L
	Alanine aminotransferase	11	7–56 U/L
	Alkaline phosphatase	60	44–147 U/L
	Total bilirubin	0.3	0.1–1.2 mg/dL
	Glomerular filtration rate non-African American	>60	≥60 mL/minute/m^2^
	Lipase	13	10–140 U/L

**Figure 1:**
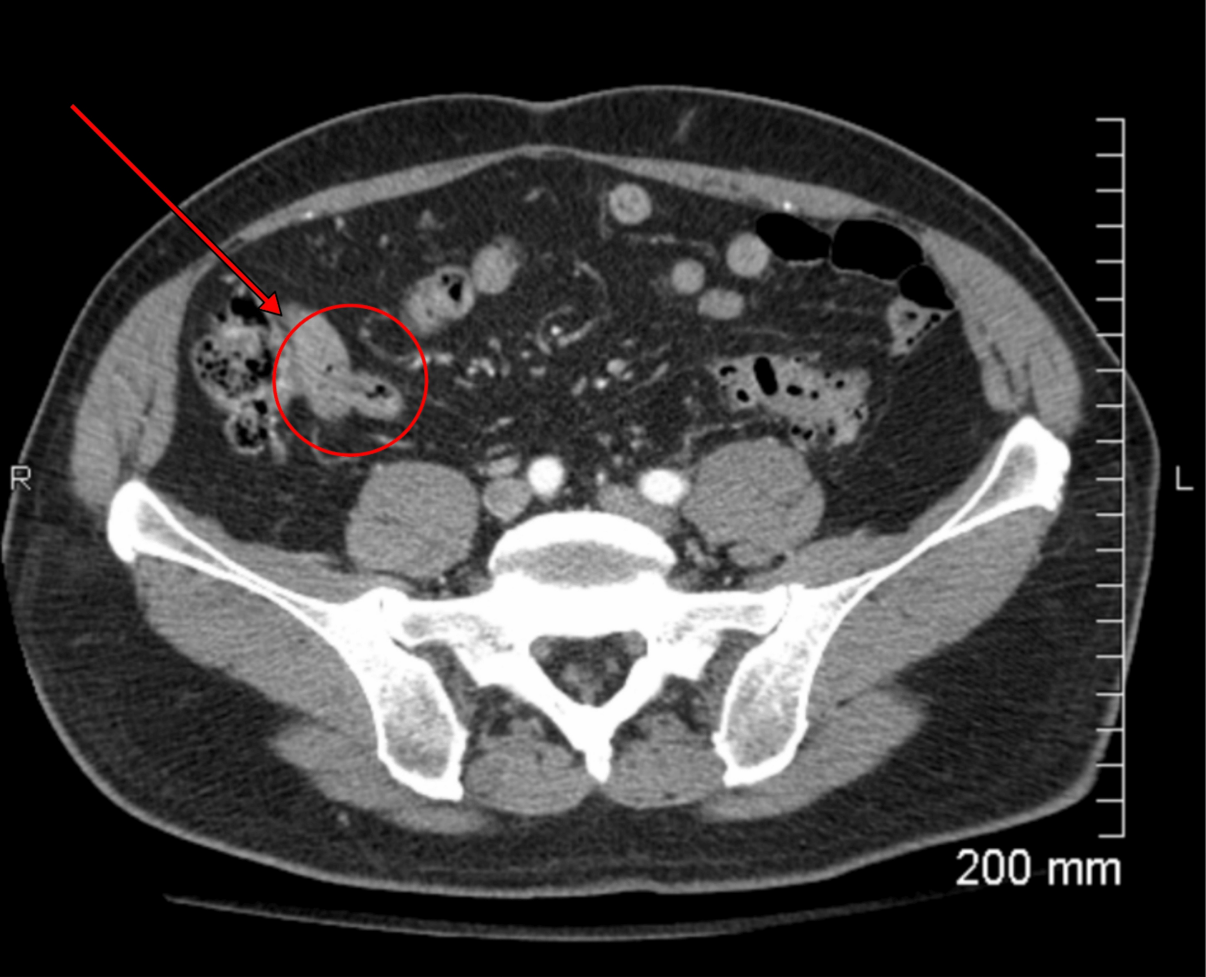
Contrast-enhanced CT of the abdomen and pelvis. Contrast-enhanced CT of the abdomen and pelvis demonstrating an elongated, retrocecal appendix measuring approximately 9.1 mm in diameter, containing an obstructing appendicolith with surrounding inflammatory changes, findings consistent with acute appendicitis. The area of interest is denoted by the red arrow.

Contrast-enhanced CT of the abdomen and pelvis revealed an elongated, retrocecal appendix extending superiorly toward the left upper abdomen, providing an anatomic explanation for the patient’s atypical pain localization. The appendix was mildly dilated, measuring up to 9.1 mm in diameter, and contained an obstructing appendicolith with associated periappendiceal inflammatory changes. No evidence of perforation, abscess formation, or free intraperitoneal air was identified. These findings confirmed the diagnosis of acute appendicitis and supported prompt surgical intervention (Figure 1).

**Figure 2:**
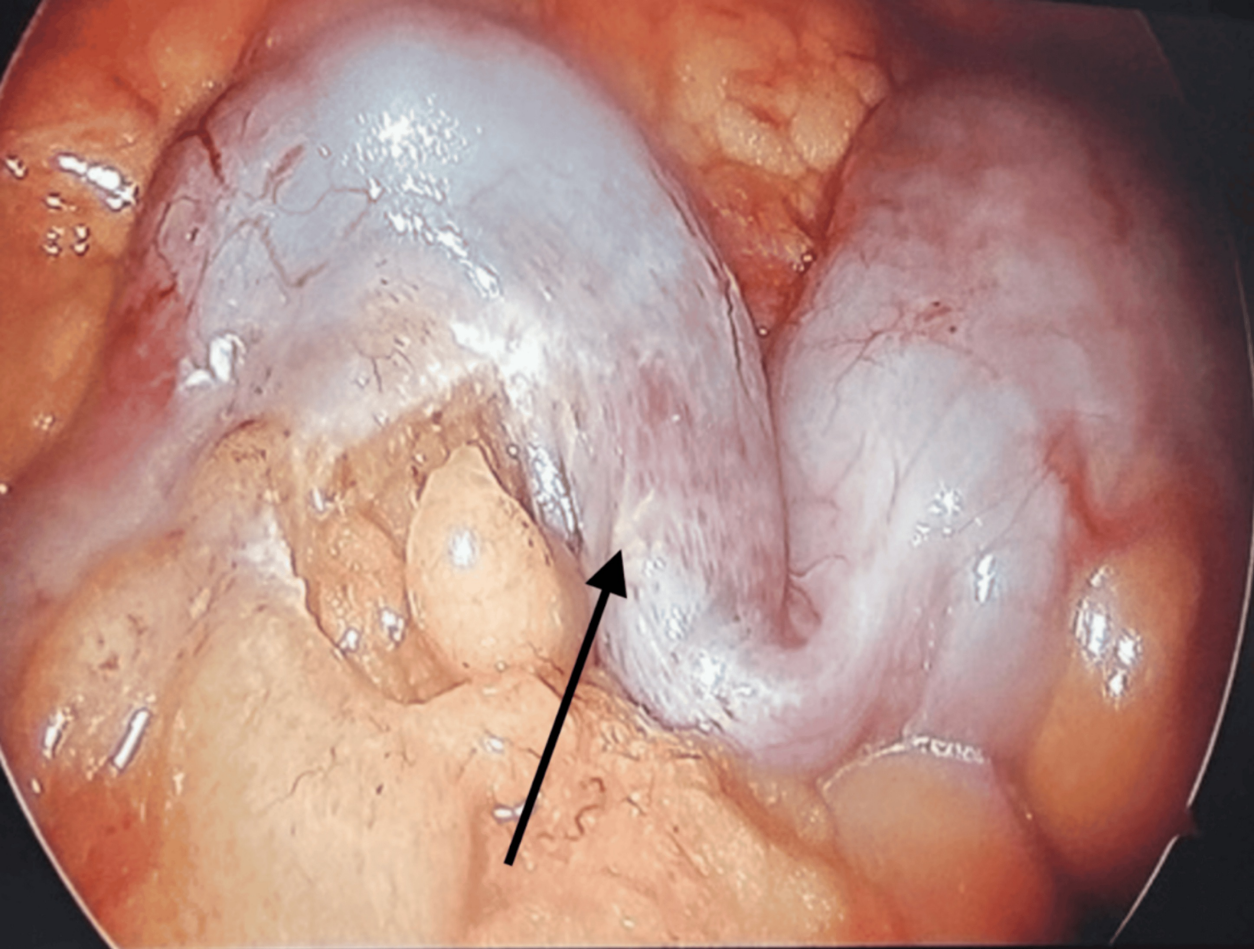
Intraoperative laparoscopic image of the appendix. The appendix is markedly inflamed and edematous (arrow) before excision. Surrounding mesoappendiceal fat appears hyperemic, consistent with acute appendicitis in a retrocecal position.

**Figure 3:**
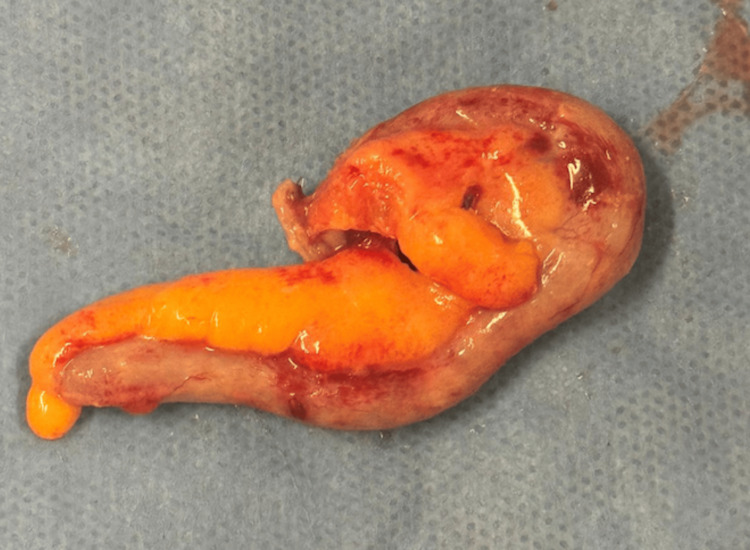
Gross specimen of the excised appendix. The excised appendix measures 8.7 cm in length and demonstrates marked edema and hyperemia of the wall, consistent with acute appendicitis. The mucosal surface shows no perforation or necrosis. No scale was included at the time of image acquisition.

The patient was taken emergently to the operating room for a laparoscopic appendectomy. Intraoperative inspection revealed a markedly inflamed, elongated appendix in a retrocecal position, consistent with the preoperative imaging findings. As depicted in Figure 2, the appendix was intact, without evidence of perforation or abscess formation. Surrounding mesoappendiceal fat was edematous and hyperemic, and minimal serosanguinous fluid was noted. The procedure was completed without intraoperative complications, and the excised specimen (Figure 3) was submitted for pathological analysis.

**Figure 4:**
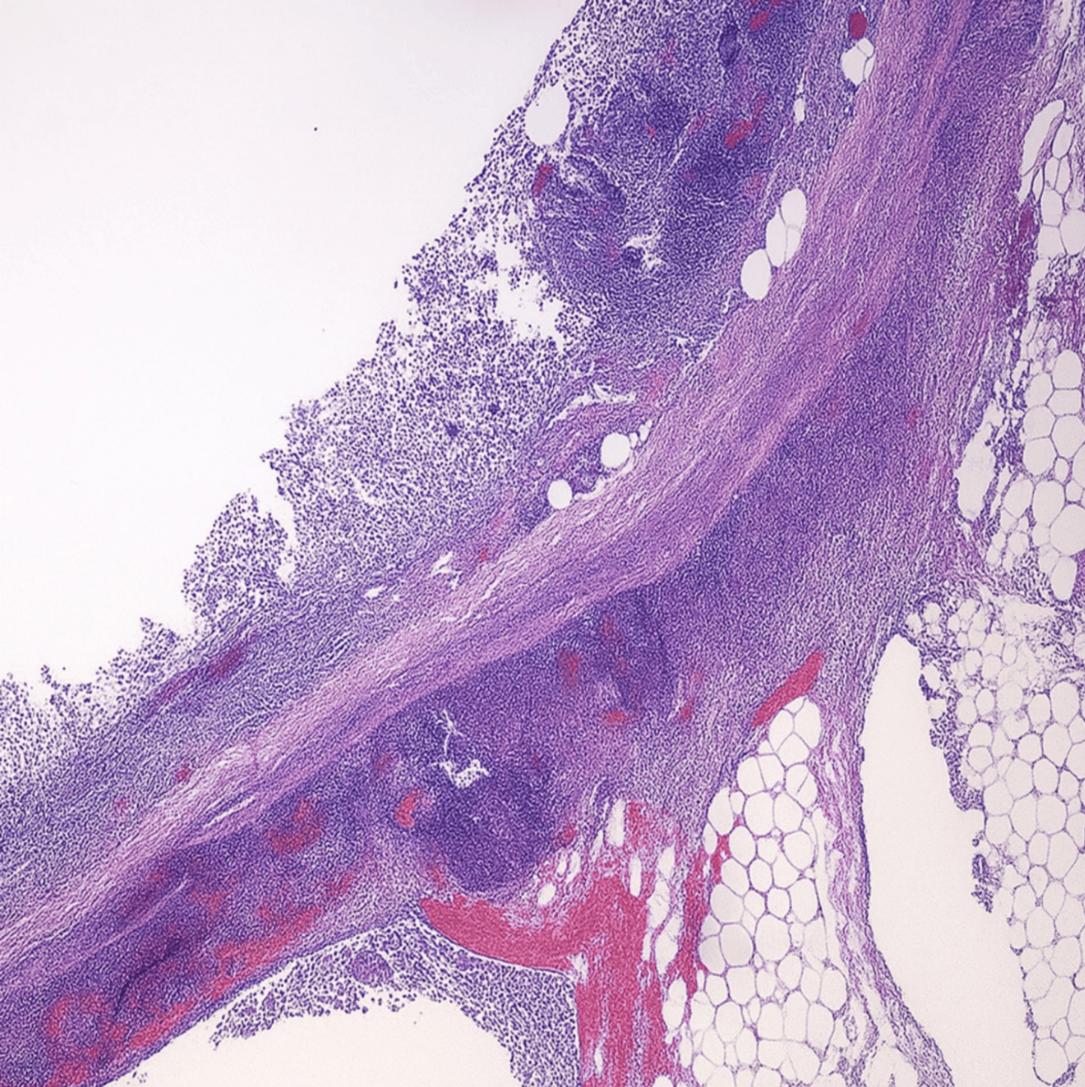
Hematoxylin and eosin-stained section of the resected appendix (10× magnification). The mucosa and submucosa are intact, with transmural infiltration of inflammatory cells consistent with acute appendicitis. The serosal surface remains smooth and intact, with no evidence of perforation, abscess formation, or necrosis. These findings corroborate the gross intraoperative appearance and confirm the clinical diagnosis.

Tissue samples from the excised appendix were submitted for pathological analysis. Examination revealed an 8.7-cm appendix with a smooth, hyperemic serosal surface. The lumen contained red, hemorrhagic fluid and inspissated fecal material. Microscopic evaluation demonstrated transmural acute inflammatory infiltrates consistent with acute appendicitis. No perforation, abscess formation, necrosis, or neoplastic lesions were identified, corroborating the clinical and intraoperative findings (Figure 4).

The patient’s postoperative course was unremarkable. She tolerated oral intake within five hours of surgery and ambulated without difficulty. Pain was well controlled with oral analgesics, and no postoperative complications were observed. She was discharged approximately 24 hours after the procedure. At her two-week follow-up, she remained asymptomatic, demonstrating complete recovery with standard wound healing and return to baseline activity.

## Discussion

This case describes a 21-year-old woman with no significant past medical history who presented with acute left upper quadrant abdominal pain, an atypical presentation for appendicitis that posed a diagnostic challenge. Imaging and intraoperative evaluation identified a retrocecal appendix containing an appendicolith. The appendix was markedly inflamed but intact, with minimal serous fluid in the pelvis and upper abdomen, and no abnormalities of the surrounding pelvic organs.

The clinical significance of this case lies in the combination of unusual pain localization and a high-risk anatomic variant. Appendiceal position strongly influences clinical presentation. While most appendices are intraperitoneal in a descending orientation, retrocecal appendices account for 26-65% of cases and are frequently associated with atypical pain patterns [3-5]. Retrocecal appendices, particularly when elongated or positioned high in the abdomen, may produce pain that deviates from the classic right lower quadrant, increasing the risk of misdiagnosis or delayed treatment [3,4]. CT imaging studies demonstrate substantial variation in appendiceal orientation, including high or posterior positions, which can obscure classic findings and contribute to diagnostic delays [6]. In such cases, reliance solely on symptom-based evaluation may lead to inappropriate reassurance or unnecessary investigations for alternative etiologies, including gastritis, renal colic, pancreatitis, or splenic pathology.

Luminal obstruction, often from a fecalith, leads to retained secretions, bacterial overgrowth, and progressive distension, typically involving *Escherichia coli* and *Bacteroides fragilis* [7]. Retrocecal inflammation primarily irritates visceral peritoneal fibers rather than parietal fibers, producing poorly localized or misleading pain [6]. Visceral afferents from the appendix travel via T10-T12 splanchnic pathways, which also carry input from the upper abdomen [6,7]. Convergence of visceral and somatic afferents in the spinal cord can cause referred pain to the left upper quadrant, flank, epigastrium, or chest, explaining the absence of classic right lower quadrant tenderness [8,9].

Atypical appendicitis presentations, such as pain localized to the left upper quadrant, often prompt consideration of a broad differential diagnosis (Table 2) and CT imaging, due to their deviation from classic right lower quadrant findings [10-12]. Gastrointestinal causes, including gastritis or peptic ulcer disease, may present with epigastric discomfort, nausea, or anorexia; however, the absence of upper gastrointestinal bleeding, history of nonsteroidal anti-inflammatory drug use, and lack of epigastric tenderness made these less likely in this patient [11,13]. Renal colic from ureterolithiasis can manifest as flank or upper quadrant pain and may be associated with hematuria or dysuria, both of which were absent [8,10,13]. Splenic pathology, such as infarction or rupture, can produce left upper quadrant pain and peritoneal signs, but there was no history of trauma, splenomegaly, or systemic symptoms [3-5,10]. Pancreatitis was considered; however, normal serum lipase levels and the absence of epigastric tenderness radiating to the back argued against this etiology [13,14]. Thoracic or pulmonary processes, including pneumonia or pleuritis, were unlikely given normal cardiopulmonary examination and the absence of cough, fever, or dyspnea [14,15].

**Table 2:** Differential diagnoses for left upper quadrant abdominal pain in atypical appendicitis. This table summarizes common non-appendiceal etiologies considered in patients presenting with left upper quadrant abdominal pain, highlighting their typical clinical features, pertinent negatives in the current patient, and relevance to the case. The comparison demonstrates how an atypical retrocecal appendicitis may mimic other conditions, underscoring the importance of imaging and careful clinical assessment for accurate diagnosis [3-5,8,10,11,13-15].

Differential diagnosis	Typical clinical features	Pertinent negatives in this patient	Relevance to the case
Gastrointestinal (gastritis, peptic ulcer disease)	Epigastric discomfort, nausea, anorexia; may have vomiting, melena, or hematemesis; often related to nonsteroidal anti-inflammatory drug use or *H. pylori* infection	No upper gastrointestinal bleeding, no history of nonsteroidal anti-inflammatory drug use, no epigastric tenderness	Atypical epigastric pain could mimic left upper quadrant presentation, but findings were inconsistent with gastrointestinal pathology [11,13]
Renal colic (ureterolithiasis)	Flank or upper quadrant pain, often radiating to the groin; may have hematuria, dysuria, nausea	No hematuria, no dysuria, pain localized to the left upper quadrant	Flank or upper quadrant pain can overlap with retrocecal appendicitis referred pain [8,10,13]
Splenic pathology (infarction, rupture)	Left upper quadrant pain, peritoneal signs, possibly hypotension; history of trauma or hematologic disorders	No trauma, no splenomegaly, no systemic symptoms	Left upper quadrant pain can mimic retrocecal appendicitis, but imaging and exam ruled this out [3-5]
Pancreatitis	Epigastric pain radiating to the back, nausea, vomiting; elevated serum lipase/amylase	Normal lipase, no epigastric tenderness radiating to the back	Important to exclude given epigastric/upper quadrant pain [13,14]
Thoracic/pulmonary (pneumonia, pleuritis)	Chest pain, cough, dyspnea, fever, abnormal breath sounds	Normal cardiopulmonary exam, no cough, fever, or dyspnea	Thoracic causes can refer pain to the upper abdomen, but were inconsistent with exam findings [14,15]

In this context, cross-sectional imaging was crucial. Contrast-enhanced CT of the abdomen and pelvis not only confirmed a dilated retrocecal appendix with an obstructing appendicolith and surrounding inflammatory changes but also excluded alternative intra-abdominal and retroperitoneal pathology, thereby definitively establishing the diagnosis [10-13]. Recent reports from 2020 to 2024 describe retrocecal or subhepatic appendicitis presenting with epigastric, flank, or thoracic pain, frequently misattributed to non-surgical etiologies until imaging clarified the diagnosis [2,9-11]. This underscores the importance of maintaining a broad differential when abdominal pain is discordant with expected anatomic patterns.

While nonoperative management with antibiotics has been increasingly considered for selected cases of uncomplicated appendicitis, particularly following the publication of the CODA trial [16], its applicability depends on patient-specific factors and anatomic considerations. The presence of an appendicolith is a well-established predictor of treatment failure, with higher rates of recurrent appendicitis, complications, and subsequent need for appendectomy compared with patients managed surgically [11,16]. In the current case, the appendicolith identified on CT, combined with the retrocecal and elongated anatomy, increased the risk of luminal obstruction and progression to perforation or abscess formation if conservative management were attempted [6,12]. Consequently, an urgent laparoscopic appendectomy was indicated to provide definitive treatment, reduce the likelihood of complications, and ensure rapid recovery. This decision highlights the importance of integrating imaging findings, anatomic risk factors, and evidence-based guidelines in selecting the optimal management strategy for atypical presentations of acute appendicitis [10-16].

This case highlights the impact of anatomical variation and neuroanatomical referral pathways on the presentation of atypical pain in appendicitis. Recognition of these variants, coupled with the judicious use of cross-sectional imaging, is crucial to prevent diagnostic delays and facilitate timely, definitive surgical management.

## Conclusions

This case illustrates the diagnostic complexity of acute appendicitis presenting with atypical pain due to a retrocecal, elongated appendix containing an appendicolith. The patient’s left upper quadrant discomfort, absence of classic right lower quadrant tenderness, and otherwise benign abdominal and cardiopulmonary examination highlight how anatomical variation can obscure traditional clinical findings. Cross-sectional imaging with CT was essential in establishing the diagnosis by demonstrating appendiceal inflammation and an obstructing appendicolith, allowing for a timely laparoscopic appendectomy. Intraoperative findings confirmed the CT results and provided definitive treatment, while postoperative recovery was rapid and uncomplicated. This case underscores the importance of maintaining a high index of suspicion for appendicitis in patients with nonclassical pain patterns, integrating imaging findings with clinical evaluation, and recognizing anatomical variants to guide prompt, individualized surgical management and minimize the risk of complications.
